# RS2910164 polymorphism in the microrna-146a is associated with risk for type 1 diabetes mellitus

**DOI:** 10.1186/1758-5996-7-S1-A205

**Published:** 2015-11-11

**Authors:** Tais Silveira Assmann, Victoria Maron Giudice, Juliana Rocha Lima, Guilherme Coutinho Kullmann Duarte, Luis Henrique Canani, Daisy Crispim

**Affiliations:** 1 Hospital de Clínicas de Porto Alegre, Porto Alegre, Brazil

## Background

Type 1 diabetes mellitus (T1D) is characterized by severe autoimmune destruction of pancreatic beta-cells, which renders subjects insulin-dependent for life. The triggering of autoimmunity against beta-cells is probably caused by a combination of environmental and genetic risk factors. Even though much is known about the genetic of T1D, more information is needed to completely unravel this tangled web. MicroRNAs (miRNAs) are a class of small noncoding RNAs molecules that negatively regulate gene expression by inducing target mRNA cleavage or by inhibiting protein translation. Abnormal miRNA expressions have been described in several pathological conditions, including autoimmune diseases. Single nucleotide polymorphisms (SNPs) in genes codifying miRNAs may alter the expression of the corresponding miRNA and, thus, confer susceptibility for a given disease. In this context, two SNPs in the miR-146a gene, rs2910164 and rs57095329, have been reported as being associated with autoimmune diseases by altering the expression of the mature miR-146a, a miRNA involved in both innate and adaptive immunity.

## Objective

To investigate whether rs2910164 SNP in miR-146a is associated with T1D.

## Materials and methods

Frequencies of the miR-146a rs2910164 (G/C) SNP were analyzed in 407 T1D patients (cases) and in 338 healthy blood-donor subjects (controls). All patients underwent physical and laboratory evaluations. The local ethics committee approved the protocol, and all patients signed an informed consent form. The rs2910164 SNP was genotype by allelic discrimination – Real-Time PCR technique using TaqMan MGB probes (Life Technologies).

## Results

Genotypes were in Hardy–Weinberg equilibrium in both samples (P=0.201).The frequency of the minor allele C was 25.7% in non-diabetic subjects and 33.9% in T1D patients (P=0.010). Moreover, the presence of the C allele in a dominant model of inheritance (CC+CG vs. GG) was associated with risk for T1D (OR=2.010; 95% CI 1.35-3.94, P=0.028), after adjustment for gender and ethnicity.

## Conclusion

The miR-146a rs2910164 SNP seem to be associated with risk for T1D. However, larger studies are necessary to confirm the association.

